# Targeting GPR68 Alleviates Inflammation and Lipid Accumulation in Metabolic Dysfunction-Associated Steatohepatitis

**DOI:** 10.3390/biology15030233

**Published:** 2026-01-26

**Authors:** Jianlei Wei, Le Wang, Zebin Mao, Pengxia Zhang

**Affiliations:** 1 Key Laboratory of Microecology-Immune Regulatory Network and Related Diseases, School of Basic Medicine, Jiamusi University, Jiamusi 154000, China; wjl17686266799@163.com; 2 Department of Biochemistry and Molecular Biology, School of Basic Medical Sciences, Peking University, Beijing 100191, China; 3 Department of Clinical Laboratory, Peking University First Hospital, Beijing 100034, China; wangle@fuwai.com

**Keywords:** GPR68, MASH, inflammation, lipid accumulation, acidification

## Abstract

Metabolic dysfunction-associated steatohepatitis (MASH) is a prevalent chronic liver disease characterized by hepatocellular injury, inflammation, and lipid accumulation, for which effective pharmacotherapies remain limited. Tissue acidification has emerged as a critical inflammatory cue in metabolic diseases; however, whether proton-sensing signaling contributes to MASH pathogenesis is largely unknown. Here, we identify the proton-sensing G protein-coupled receptor GPR68 (OGR1) as a key inflammatory and metabolic regulator in diet-induced MASH. In a high-fat diet-induced mouse model, hepatic GPR68 expression was markedly upregulated during MASH progression. Inhibiting GPR68 with ogremorphin significantly ameliorated MASH pathology, as evidenced by reduced hepatic inflammation and lipid accumulation, improved histological features, and attenuated expression and release of pro-inflammatory cytokines, including IL-6 and TNF-α. Mechanistically, GPR68 inhibition dampened acidification-associated inflammatory signaling in the liver. Together, these findings establish GPR68 as a tractable proton-sensing inflammatory node that links tissue acidification to metabolic liver injury and highlight GPR68 as a promising therapeutic strategy for MASH.

## Simple Summary

Metabolic dysfunction-associated steatohepatitis (MASH) is a common chronic liver disease for which effective drug treatments are still lacking. In this study, we show that GPR68, a receptor that senses acidic changes in tissues, is abnormally increased in the liver during MASH progression. Blocking GPR68 activity reduced liver inflammation and fat accumulation in a diet-induced mouse model of MASH. These findings suggest that targeting acid-sensing pathways, such as GPR68, may offer a new therapeutic approach for MASH.

## 1. Introduction

According to epidemiological and modeling studies, metabolic dysfunction-associated steatohepatitis (MASH) is estimated to affect approximately 3–5% of the adult population worldwide [1]. In the United States, the number of individuals with MASH is projected to increase from 14.9 million to 23.2 million by 2050 [2], and similar upward trends are being observed globally, particularly among high-risk populations such as individuals with obesity and type 2 diabetes mellitus. Without effective intervention, MASH can progress to advanced liver disease, including cirrhosis, hepatocellular carcinoma, and liver-related mortality, as demonstrated by long-term longitudinal studies [3]. Despite its growing clinical burden, therapeutic options for MASH remain limited. The recent regulatory approval of resmetirom represents an important milestone in the field [4]. In parallel, metabolic agents such as semaglutide have shown promise in improving metabolic and histological features of MASH and are being actively evaluated in ongoing clinical studies [5]. However, the failure of multiple previously promising candidates in phase III clinical trials underscores the substantial challenges in MASH drug development [6]. Collectively, these observations highlight an urgent need to identify novel, safe, and effective therapeutic strategies for MASH.

Local tissue acidification is a recognized feature of inflammatory diseases and has been shown to actively modulate immune and inflammatory signaling pathways through pH-sensitive mechanisms [7]. Although direct measurements of hepatic tissue pH in patients with MASH are currently lacking, experimental models of MASH exhibit hypoxia-associated metabolic remodeling, including activation of hypoxia-inducible factor-1α and altered lactate metabolism, suggesting the presence of a locally perturbed, potentially acidified hepatic microenvironment [8]. Importantly, inflammation and endoplasmic reticulum stress, both central drivers of MASH pathogenesis, are tightly linked to metabolic stress responses, and accumulating evidence indicates that cellular stress signaling can be modulated by pH-dependent regulation of gene expression [9]. Together, these findings raise the possibility that tissue acidification may function as an amplifying microenvironmental cue in MASH, providing a mechanistic rationale for targeting proton-sensing pathways such as GPR68 [10].

Given the emerging role of acidification in inflammatory signaling, pH-sensing receptors represent attractive therapeutic targets. G protein-coupled receptors (GPCRs) constitute one of the most druggable receptor families, characterized by diverse physiological functions, finely tunable signal transduction, and a high success rate in clinical drug development [11,12]. Notably, recent studies have begun to identify specific GPCRs as functionally relevant and druggable targets in MASH, highlighting the emerging therapeutic potential of GPCR-directed strategies [13]. Among these, proton-sensing GPCRs have been implicated in coordinating inflammatory responses and metabolic reprogramming in acidic microenvironments. Supporting this concept, pharmacological inhibition of the proton-sensing receptor GPR68 significantly alleviated inflammation and reduced macrophage infiltration in a dextran sulfate sodium-induced colitis model, accompanied by decreased secretion of IL-6 and TNF-α [14]. Importantly, independent evidence from liver-focused studies has demonstrated that modulation of inflammatory signaling pathways is sufficient to ameliorate hepatic steatosis and inflammatory injury. In this regard, bioactive compounds with anti-inflammatory properties have been shown to confer hepatoprotective effects by attenuating inflammatory signaling cascades and metabolic stress responses [15]. Although colitis and MASH differ in etiology, they share key inflammatory mediators, suggesting that GPR68 may represent a functionally relevant inflammatory node across distinct disease contexts.

In this study, we employed integrated in vitro and in vivo approaches to investigate the role of GPR68 in MASH. We demonstrate that GPR68 expression is markedly upregulated in experimental models of fatty liver disease and MASH. Functional studies reveal that GPR68 regulates the production of pro-inflammatory cytokines, including IL-6 and TNF-α, thereby contributing to disease progression. Furthermore, treatment with the small-molecule GPR68 inhibitor ogremorphin (OGM) significantly attenuated MASH pathology in a diet-induced mouse model. Collectively, our findings identify GPR68 as a previously underappreciated regulator of hepatic inflammation and establish pharmacological inhibition of this proton-sensing receptor as a potential therapeutic strategy for MASH.

## 2. Materials and Methods

### 2.1. Ethics Statement

All animal experiments were approved by the Animal Ethics and Use Committee of Jiamusi University Medical College (Approval No. JDJCYXY20250068). All animal husbandry and experimentation strictly adhered to the institution’s guidelines for the care and use of laboratory animals.

### 2.2. Animals and Models

Male ApoE^−^/^−^ mice (C57BL/6 background), 8 weeks of age, were purchased in two batches from Peking University Health Science Center. The first batch (*n* = 12) was used for MASH model validation: mice were randomly assigned to a normal control group (NC; *n* = 6) or a high-fat diet group (HFD; *n* = 6). The standard chow diet and HFD were obtained from Nanjing Synergy Biosciences (Nanjing, China), with catalog numbers XTAA (A10021B) and XTM05-005 (XT310-1), respectively. The HFD provided 41.4% of total energy from fat, 39.9% from carbohydrates (including 20% fructose), and 18.8% from protein, and was supplemented with 2% cholesterol. Mice were fed for 16 weeks, after which tissues and blood were collected to confirm successful model establishment. The second batch (*n* = 18) was used for OGM intervention studies: after a one-week acclimation period, mice were randomly assigned to three groups: (1) NC (*n* = 6), (2) HFD + vehicle (*n* = 6), and (3) HFD + OGM (*n* = 6). All mice were housed under specific pathogen-free conditions (22 ± 2 °C, 45% ± 10% humidity, 12-h light-dark cycle) with free access to food and water. Mice in the HFD + OGM group received daily oral gavage of 30 mg/kg OGM (HY-154954, MCE, Monmouth Junction, NJ, USA) starting from week 8 of the HFD, while control and HFD + vehicle groups received the equivalent vehicle. The OGM dosage was selected based on a previously published in vivo study demonstrating effective GPR68 inhibition and good tolerability [16], despite being conducted in a different disease model, and was further refined by our preliminary dose-ranging experiments. In these experiments, 10 mg/kg produced limited therapeutic effects, whereas 30 mg/kg achieved efficacy comparable to higher doses without additional benefit. Therefore, 30 mg/kg was chosen as an effective and appropriate dose for subsequent intervention studies. After 8 weeks of treatment (week 16 of the diet), mice were euthanized and tissue and blood samples were collected for subsequent data analysis.

### 2.3. Cell Culture and Treatment

HepG2 cells were obtained from the laboratory of Professor Mao Zebin at Peking University Health Science Center (Beijing, China). The cells were cultured in high-glucose Dulbecco’s modified Eagle medium (DMEM, KeyGEN BioTECH, Nanjing, China) supplemented with 10% fetal bovine serum (FBS, Gibco, Waltham, MA, USA) and 1% penicillin-streptomycin (Sangon, Shanghai, China) at 37 °C and 5% CO_2_. To establish an in vitro steatosis model, cells were treated for 24 h with an oleic acid/palmitic acid mixture at a molar ratio of 2:1 (total concentration, 750 μM), consisting of 500 μM oleic acid (OA, sodium oleate, TargetMol, Shanghai, China) and 250 μM palmitic acid (PA, sodium palmitate, MCE, Princeton, NJ, USA), conjugated with fatty acid-free BSA (final BSA concentration, 0.5%). Control cells received culture medium containing the same concentration of fatty acid-free BSA without fatty acids. For OGM treatment, HepG2 cells were treated with OGM at a final concentration of 2.5 μM. This concentration was determined based on preliminary dose-ranging experiments (1–10 μM), in which 2.5 μM exhibited optimal efficacy without apparent cytotoxicity. Cells were treated with OGM at a final concentration of 2.5 μM for 0, 6, 12, or 24 h in the presence of a mixture of oleic acid and palmitic acid (OA/PA). OGM was dissolved in DMSO, and control cells were treated with an equivalent volume of vehicle (0.1% DMSO).

### 2.4. Antibodies and Reagents

The antibodies used in this study were: GPR68 (mouse/human, ABclonal, Woburn, MA, USA, A21838), IL-6 (mouse, Thermo, Waltham, MA, USA, 88-7064; human, Thermo, 88-7066), TNF-α (mouse, Thermo, 88-7324; human, Thermo, 88-7346), and β-actin (mouse/human, ZSGBio, Beijing, China, TA-09). Detailed information on other compounds used in this study and their manufacturers is as follows: ogremorphin (MCE, HY-154954), sodium oleate (TargetMol, T36390), and sodium palmitate (MCE, HY-N0830B).

### 2.5. siRNA-Mediated Knockdown of GPR68

For human GPR68 knockdown, three independent siRNAs (3-oligo pool) were designed and synthesized. The sequence exhibiting the highest silencing efficiency (>80% reduction at 48 h) was used for all subsequent experiments. Human GPR68 siRNA (sense, 5′-CCACCGUUGUCACAGACAAUGTT-3′) and a scrambled negative control were synthesized by RuiBiotech (Beijing, China). HepG2 cells were seeded in 6-well plates at 2 × 10^5^ cells/well and transfected with 50 nM siRNA using Vigofect transfection reagent (Vigorous Biotechnology, Beijing, China) according to the manufacturer’s instructions. After 6 h, the medium was replaced with fresh complete medium. Gene-silencing efficiency was assessed 48 h post-transfection by WB and RT-qPCR, followed by 24 h OA/PA treatment.

### 2.6. Blood Analysis

For routine blood tests, 60 μL of fresh blood was collected from each mouse and transferred immediately into an EDTA tube. Samples were analyzed with an automated hematology analyzer (Mindray, Shenzhen, China, BC-2800Vet). For serum biochemical analysis, blood was collected, allowed to clot at 37 °C for 50 min, and then stored at 4 °C overnight. Serum was obtained by centrifugation at 1200× *g* for 12 min. Serum samples (220 μL) were analyzed for total cholesterol (TC), triglycerides (TG), alanine aminotransferase (ALT) and aspartate aminotransferase (AST) using a chemical analyzer (Mindray, Chemray 800).

### 2.7. Histological Analysis

#### 2.7.1. Hematoxylin and Eosin (H&E) Staining

Paraffin-embedded liver sections (4 μm) were deparaffinized, rehydrated, and stained with hematoxylin and eosin using standard protocols. Images were acquired with an Olympus BX53 microscope (Olympus, Tokyo, Japan).

#### 2.7.2. Immunohistochemical (IHC) Staining

Mouse liver tissues were excised, fixed overnight in 4% paraformaldehyde, paraffin-embedded, and cut into 5-µm-thick sections. Sections underwent hematoxylin–eosin staining followed by immunohistochemical (IHC) detection of GPR68 expression. ImageJ (version 1.53, National Institutes of Health, Bethesda, MD, USA) was used for quantitative analysis of GPR68-positive areas. The IHC procedure followed standard protocols using a primary antibody against GPR68 (ABclonal, A21838).

### 2.8. Oil Red O Staining

#### 2.8.1. Liver Tissue

Oil Red O staining was used to assess hepatic steatosis. Mouse liver tissues were snap-frozen in dry ice, OCT-embedded, and cut into 8-µm sections. After fixation in 4% paraformaldehyde for 10 min, sections were rinsed in 60% isopropanol for 20 s and incubated with 0.5% Oil Red O (in 60% isopropanol) at 37 °C for 15 min. After washing with 60% isopropanol for 1 min and rinsing briefly in double-distilled water, slides were mounted with glycerol–gelatin. Whole-slide images were acquired with a 3DHISTECH P250FLASH scanner (3DHISTECH Ltd., Budapest, Hungary) and quantified using ImageJ.

#### 2.8.2. Cells

Intracellular lipid accumulation was quantified by Oil Red O staining. Cells were seeded in 24-well plates, treated with OA/PA for 24 h, washed twice with PBS, and fixed with 4% paraformaldehyde for 10 min. After 20 s of immersion in 60% isopropanol, cells were incubated with 0.5% Oil Red O working solution at 37 °C for 15 min. After washing in 60% isopropanol for 1 min and a brief rinse in distilled water, images were acquired with a digital microscope and analyzed with ImageJ.

#### 2.8.3. ELISA Analysis

Serum for ELISA was prepared as described in the Section 2.6. Serum levels of IL-6 (Thermo, 88-7064) and TNF-α (Thermo, 88-7324) were measured using specific ELISA kits. After OA/PA treatment for 24 h, HepG2 cell culture supernatant was collected and centrifuged at 300× *g* for 5 min to remove cellular debris. Levels of IL-6 (Thermo, 88-7066) and TNF-α (Thermo, 88-7346) were measured using specific ELISA kits.

#### 2.8.4. CCK-8 Assay

Cell viability was assessed using the CCK-8 assay. Cells (4 × 10^3^ cells/well) were seeded in 96-well plates with 100 μL medium per well. After attachment, drugs were added as indicated. After treatment, CCK-8 reagent was added at a final concentration of 10% (Dojindo, Kumamoto, Japan), and the plates were incubated at 37 °C for 1 h. Absorbance at 450 nm was measured using a microplate reader (Thermo Fisher Scientific, Waltham, MA, USA).

#### 2.8.5. RNA Extraction and RT-qPCR Analysis

Total RNA was extracted from mouse tissues and HepG2 cells using TransZol reagent (TransGen Biotech, Beijing, China, ET101-01-V2). cDNA was synthesized via reverse transcription using TransScript II All-in-One First-Strand cDNA Synthesis SuperMix (TransGen Biotech, AH341-01). Gene expression was quantified by RT-qPCR using PerfectStart Green qPCR Master Mix (TransGen Biotech, AQ602-01). Relative expression levels were calculated using the 2^−ΔΔCt^ method. Gapdh and GAPDH served as endogenous normalization controls for mouse and human samples, respectively. Primer sequences used for RT-qPCR analysis were provided (Supplementary Table S1).

#### 2.8.6. Western Blot

Liver tissues and HepG2 cells were lysed for 20–30 min in ice-cold RIPA buffer (Bristol Myers, Princeton, NJ, USA, catalog no. P0013B) containing protease and phosphatase inhibitors (1 mM each). Lysates were centrifuged at 12,000× *g* for 15 min at 4 °C, and supernatants were collected. Protein concentration was determined using the BCA Protein Assay Kit (Becton Dickinson, Franklin Lakes, NJ, USA, P0010). Equal amounts of protein were separated by 10–12% SDS-PAGE and transferred to a 0.45 μm PVDF membrane. The membrane was blocked with 5% non-fat dry milk for 1 h and incubated overnight with the primary antibody at 4 °C. It was then incubated with the HRP-labeled IgG secondary antibody at room temperature for 1 h. Protein bands were visualized using the Ultra-Sensitive ECL Kit (Millipore, Burlington, MA, USA, P90720) and imaged with a ChemiDoc system (Bio-Rad, Hercules, CA, USA). Band intensities were quantified using ImageJ software.

#### 2.8.7. Measurement of pH in Cell-Culture Supernatant

The pH of the cell culture supernatant was measured with a pH meter (Sartorius PB-10, Göttingen, Germany). All experimental groups were cultured under identical conditions, including the same CO_2_ concentration (5%), culture volume, and initial cell seeding density. Culture medium supernatants were collected in parallel after 24 h of treatment, and pH changes reflected treatment-induced extracellular acidification rather than differences in environmental conditions.

#### 2.8.8. Statistical Analysis

Data were analyzed using GraphPad Prism 10.4. Results are expressed as mean ± SEM. Comparisons between two groups were performed using independent-samples *t*-test. Comparisons between multiple groups were performed using one-way ANOVA followed by Dunnett’s post hoc test. Comparisons between multiple groups in experiments with two categorical independent variables were performed using two-way ANOVA followed by Dunnett’s post hoc test. *n* denotes the number of mice or independent experiments. * *p* < 0.05, ** *p* < 0.01, *** *p* < 0.001; ns indicates no statistical significance (*p* > 0.05).

## 3. Results

### 3.1. GPR68 Is Upregulated in MASH

**Figure 1:**
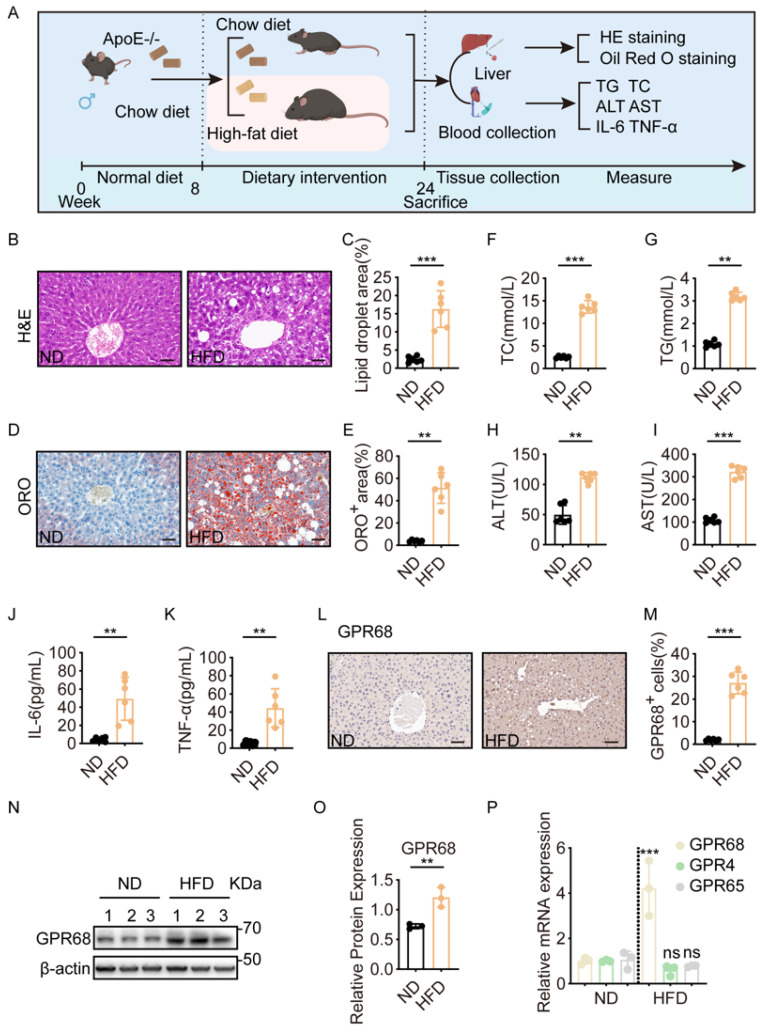
Hepatic GPR68 expression was elevated in MASH mice. (**A**–**P**) ApoE^−^/^−^ mice on a C57BL/6J background were fed a normal diet (ND) or a high-fat diet (HFD) for 16 weeks. (**A**) Schematic diagram of HFD-induced MASH model in mice. (**B**) H&E staining of mouse liver tissue. (**C**) Lipid titration analysis. (**D**) Representative Oil Red O staining of mouse liver tissue. (**E**) Quantification of ORO-positive areas in liver tissue from mice. (**F**–**K**) Serum biochemical test. The levels of (**F**) total cholesterol (TC), (**G**) triglycerides (TG), (**H**) alanine transaminase (ALT), (**I**) aspartate transaminase (AST), (**J**) IL-6, (**K**) TNF-α. (**L**) Representative IHC images of GPR68 in mouse liver sections. (**M**) Qualitative analysis of GPR68-positive cells. (**N**) GPR68 protein expression in mouse liver was detected by Western blot (WB). (**O**) Quantitative analysis of GPR68 protein, normalized to β-actin. (**P**) The mRNA expression levels of proton-sensing receptors GPR68, GPR4, and GPR65 in mouse liver were measured. Data are presented as mean ± SEM. (**B**,**D**,**L**) Scale bar, 50 μm. (**B**–**M**) *n* = 6 per group. (**N**–**P**) *n* = 3. Unpaired two-tailed *t* test for (**C**,**E**–**K**,**M**,**O**), One-way ANOVA followed by Dunnett’s test for (P), ** *p* < 0.01, *** *p* < 0.001. ns, not significant.

To investigate the relationship between GPR68 and MASH, ApoE^−^/^−^ mice were fed an HFD to induce MASH (Figure 1A). The model recapitulated core pathological features, including steatosis and inflammation. Hepatocyte ballooning and inflammatory infiltration were observed after 16 weeks of HFD feeding (Figure 1B,C), and Oil Red O staining revealed extensive lipid droplet accumulation (Figure 1D,E). Consistent with this, serum total cholesterol (TC) and triglyceride (TG) levels (Figure 1F,G), the hepatocyte injury markers alanine aminotransferase (ALT) and aspartate aminotransferase (AST) (Figure 1H,I), and serum IL-6 and TNF-α levels (Figure 1J,K) were elevated in HFD-fed mice compared with normal diet-fed mice. These data confirm the establishment of the MASH model. Next, immunohistochemical analysis revealed that GPR68 expression was significantly upregulated in the livers of HFD-fed mice (Figure 1L,M), which was further confirmed by Western blot (WB) analysis (Figure 1N,O). The corresponding original Western blot images for all Western blot experiments are provided in Supplementary Figure S1, and the densitometric analysis results are presented in Supplementary Table S2. However, the expression of two other proton-sensing receptors, GPR4 and GPR65, did not change significantly (Figure 1P), suggesting that GPR68 is specifically associated with MASH.

### 3.2. GPR68 Expression Increases in OA/PA-Induced HepG2 Cells

**Figure 2:**
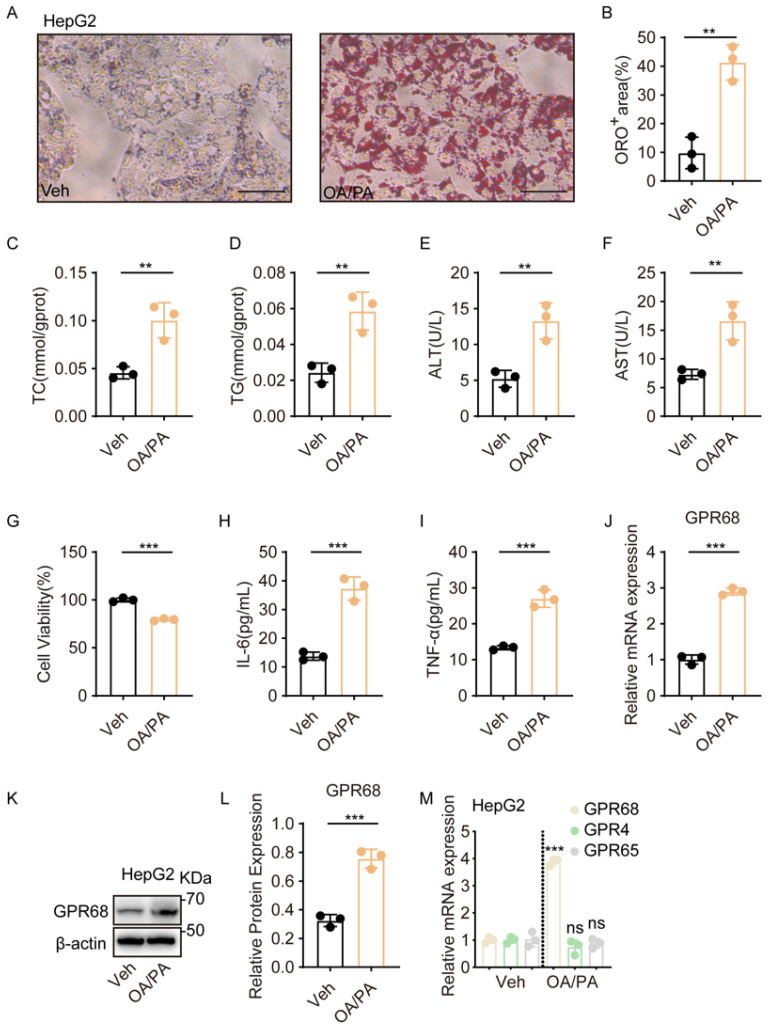
GPR68 expression was increased in OA/PA-induced steatotic HepG2 cells. (**A**–**M**) HepG2 cells were treated with an FFA mixture (OA:PA 2:1, 750 μM) for 24 h to induce steatosis. (**A**) Representative images of Oil Red O staining, Scale bar, 50 μm. (**B**) Quantitative analysis of ORO-positive regions. (**C**) Intracellular TC content. (**D**) Intracellular TG content. (**E**) ALT content in the culture medium. (**F**) AST content in the culture medium. (**G**) Quantification of viability of HepG2 cells. (**H**) IL-6 levels in cell culture supernatant. (**I**) TNF-α levels in cell culture supernatant. (**J**) GPR68 mRNA expression by quantitative reverse transcription polymerase chain reaction (RT-qPCR). (**K**) Representative WB images. (**L**) Quantitative analysis of GPR68 protein, normalized to β-actin. (**M**) The mRNA expression levels of proton-sensing receptors GPR68, GPR4, and GPR65 in HepG2 cells were subsequently determined. Data are presented as mean ± SEM. (**A**–**M**) *n* = 3. Unpaired two-tailed *t* test for (**B**–**J**,**L**), one-way ANOVA followed by Dunnett’s test for (**M**), ** *p* < 0.01, *** *p* < 0.001. ns, not significant. The dotted line indicates the separation between the Veh and OA/PA groups.

To further confirm the effect of lipid accumulation on GPR68 expression in liver cells, we established a HepG2 model using co-treatment with oleic acid (OA) and palmitic acid (PA), which is widely used to recapitulate the steatosis and injury seen in MASH. Oil Red O staining showed significantly elevated lipid deposition in OA/PA-treated HepG2 cells relative to untreated controls (Figure 2A,B). Measurements of intracellular TC and TG further confirmed that OA/PA induced lipid accumulation in HepG2 cells (Figure 2C,D). Moreover, lipid accumulation was accompanied by a moderate reduction in cell viability (Figure 2E–G). Specifically, OA/PA treatment reduced HepG2 cell viability to 79.44 ± 0.78% of the control (mean ± SEM, *n* = 3) (Figure 2G). In parallel, the expression of IL-6 and TNF-α was increased (Figure 2H,I), indicating that OA/PA treatment induced metabolic stress and inflammatory responses consistent with the in vivo findings. Next, we examined GPR68 expression in OA/PA-treated HepG2 cells. Quantitative reverse transcription polymerase chain reaction (RT-qPCR) and WB analysis revealed that GPR68 mRNA and protein levels were significantly higher than those in the controls (Figure 2J–L), whereas the expression of GPR65 and GPR4 did not differ significantly (Figure 2M). These results not only further confirm the impact of lipid accumulation on GPR68 expression, but also provide a rationale for further investigation into the function of GPR68 in MASH using this cell model.

### 3.3. Extracellular Acidification Promotes GPR68-Dependent Inflammatory Signaling in OA/PA-Induced HepG2 Cells

**Figure 3:**
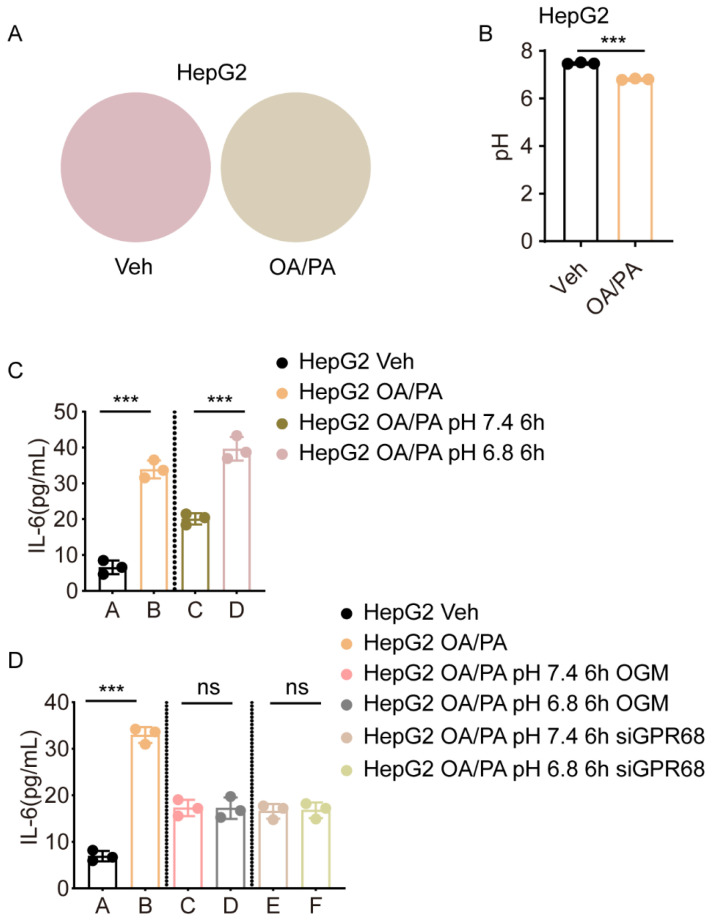
OA/PA Induces GPR68 Activity in HepG2 Cells. (**A**,**B**) HepG2 cells were treated with an FFA mixture (OA:PA 2:1, 750 μM) for 24 h to induce steatosis. (**A**) Representative images of the culture medium supernatant containing phenol red are shown, illustrating color changes from red to yellow. No cells are present in this image. (**B**) Media pH changes in cell culture supernatant. (**C**) HepG2 cells were treated with OA/PA (2:1, 750 μM) and cultured in acidic (pH 6.8) or neutral (pH 7.4) medium for 6 h and IL-6 levels in the cell culture supernatant. (**D**) HepG2 cells were treated with OA/PA (2:1, 750 μM) in the presence of OGM or siGPR68, and cultured in acidic (pH 6.8) or neutral (pH 7.4) medium for 6 h and TNF-α levels in the cell culture supernatant. Data are presented as mean ± SEM. (**A**–**D**) *n* = 3. Unpaired two-tailed *t* test for (**B**), two-way ANOVA followed by Dunnett’s test for (**C**,**D**), *** *p* < 0.001. ns, not significant.

After examining GPR68 expression, we determined whether GPR68 is activated in OA/PA-induced HepG2 cells. In this experiment, IL-6 expression was used as an indicator of GPR68 activation. It is well-known that GPR68 activation is dependent on extracellular acidification. Thus, we measured the pH of the culture medium after treating HepG2 cells with OA/PA for 24 h. The culture medium supernatant, collected after treatment, changed from red to yellow (Figure 3A), reflecting acidification of the medium. Direct pH meter measurements revealed a significant decrease in extracellular pH following OA/PA treatment (Figure 3B). To determine whether this acidification was essential for GPR68 activation, HepG2 cells treated with OA/PA for 24 h were cultured using a medium with a pH of 7.4 (representing neutrality) or a pH of 6.8 (representing acidity) for 6 h. The results showed that the medium with pH 7.4 significantly attenuated IL-6 expression compared to the medium with pH 6.8 (Figure 3C). However, GPR68 knockdown or inhibition by the GPR68 inhibitor OGM abolished the effect of medium pH on the expression of this inflammatory factor (Figure 3D). These data suggest that (1) extracellular acidification is correlated with increased GPR68 activity in OA/PA-induced HepG2 cells and (2) GPR68 activation at least partly contributes to the inflammatory response.

### 3.4. GPR68 Knockdown Alleviates OA/PA-Induced MASH-like Phenotypes in HepG2 Cells

**Figure 4:**
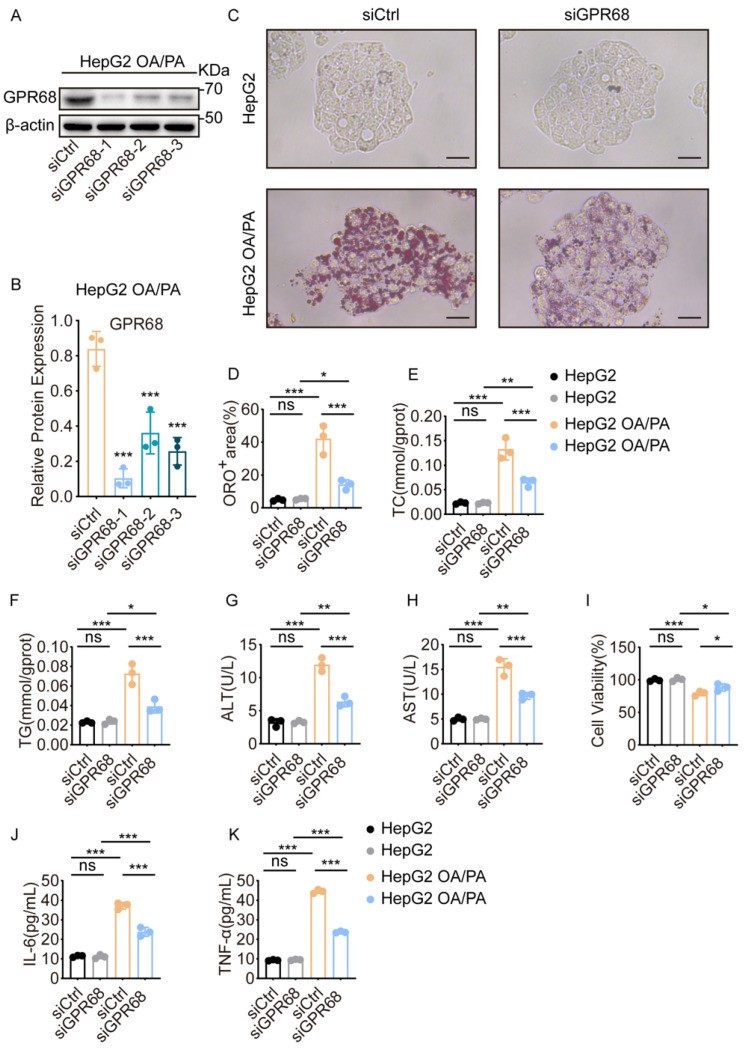
GPR68 knockdown reduced lipid accumulation and pro-inflammatory cytokine secretion in OA/PA-treated HepG2 cells. (**A**–**K**) HepG2 cells were transfected with 50 nM siCtrl or siGPR68 for 48 h, followed by OA/PA (2:1, 750 μM) treatment for 24 h. (**A**) Representative WB images. (**B**) Quantitative analysis of GPR68 protein, normalized to β-actin. (**C**) Representative images of Oil Red O staining, Scale bar, 50 μm. (**D**) Quantitative analysis of ORO-positive regions. (**E**) Intracellular TC content. (**F**) Intracellular TG content. (**G**) ALT content in the culture medium. (**H**) AST content in the culture medium. (**I**) Quantification of viability of HepG2 cells. (**J**) IL-6 levels in cell culture supernatant. (**K**) TNF-α levels in cell culture supernatant. Data are presented as mean ± SEM. (**A**–**K**) *n* = 3. One-way ANOVA followed by Dunnett’s test for (**B**), two-way ANOVA followed by Dunnett’s test for (**D**–**K**), * *p* < 0.05, ** *p* < 0.01, *** *p* < 0.001. ns, not significant.

To investigate whether GPR68 upregulation contributes to MASH, we knocked down GPR68 by transiently transfecting GPR68-specific siRNA into HepG2 cells. WB analysis showed significantly reduced GPR68 protein levels, confirming efficient knockdown (Figure 4A,B). Subsequently, we observed the effect of GPR68 knockdown on the phenotypes of OA/PA-treated HepG2 cells. Results showed that GPR68 knockdown significantly reduced lipid droplet accumulation (Figure 4C,D), intracellular TC and TG levels (Figure 4E,F), cytotoxicity as evidenced by reduced ALT/AST release and improved cell viability (Figure 4G–I), and expression of IL-6 and TNF-α (Figure 4J,K). These data suggest that GPR68 plays a key role in MASH pathogenesis.

### 3.5. Pharmacological Inhibition of GPR68 Alleviates OA/PA-Induced MASH-like Phenotypes in HepG2 Cells

**Figure 5:**
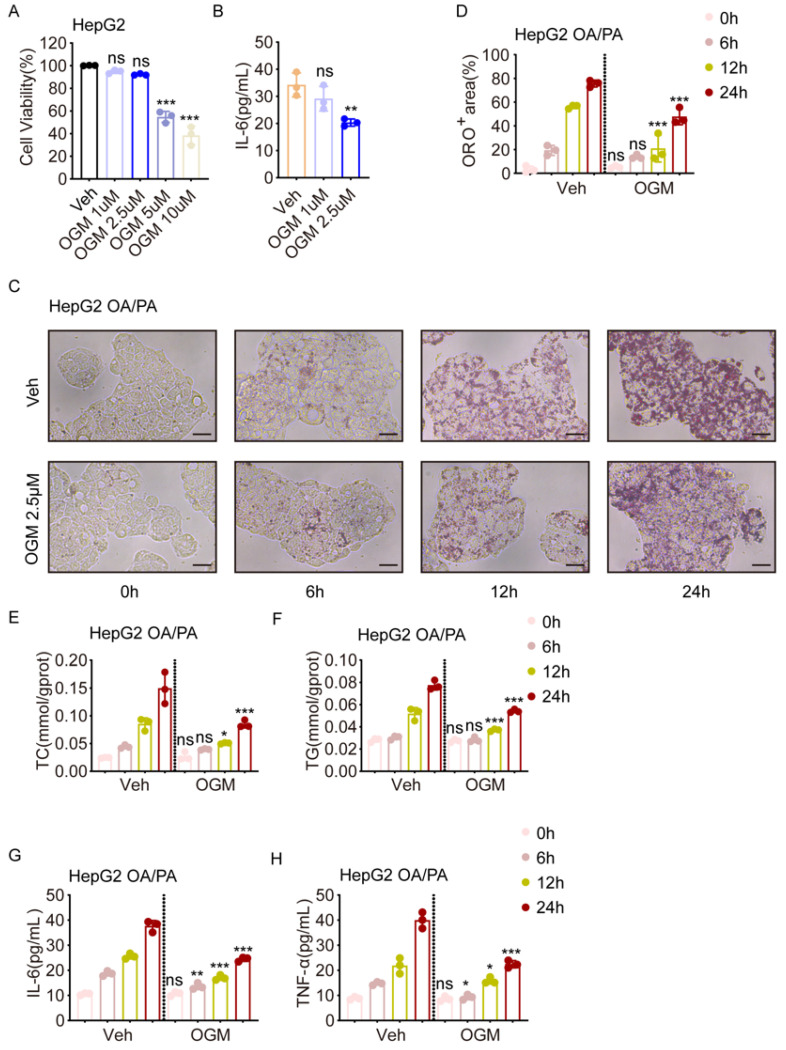
The GPR68 inhibitor OGM alleviated lipid deposition and pro-inflammatory factor expression in HepG2 cells with OA/PA-induced steatosis. (**A**) HepG2 cell viability after OGM treatment. (**B**) HepG2 cells pretreated with OA/PA were treated with different concentrations of OGM or vehicle for 24 h, and IL-6 levels in the cell culture supernatant were measured. (**C**–**H**) HepG2 cells were co-treated with OA/PA (2:1, 750 μM) and 2.5 μM OGM or vehicle (Veh) at 0, 6, 12, and 24 h. (**C**) Representative images of Oil Red O staining, scale bar, 50 μm. (**D**) Quantitative analysis of ORO-positive regions. (**E**) Intracellular TC content. (**F**) Intracellular TG content. (**G**) IL-6 levels in cell culture supernatant. (**H**) TNF-α levels in cell culture supernatant. Data are presented as mean ± SEM. (**A**–**H**) *n* = 3. One-way ANOVA followed by Dunnett’s test for (**A**,**B**), two-way ANOVA followed by Dunnett’s test for (**D**–**H**), * *p* < 0.05, ** *p* < 0.01, *** *p* < 0.001. ns, not significant.

To further confirm the above results, we treated HepG2 cells with a GPR68 inhibitor, OGM. To establish optimal experimental conditions, HepG2 cells were treated with increasing concentrations of OGM, and the CCK-8 assay was used to determine cell viability across OGM doses. The results showed that cytotoxicity became evident at concentrations above 5 μM (Figure 5A). Furthermore, we found that OGM effectively inhibited IL-6 expression at 2.5 μM, but not at 1 μM. Thus, we chose 2.5 μM for the following experiments (Figure 5B). We first observed the effect of OGM on lipid accumulation at the indicated time points. The results showed that OGM treatment reduced lipid accumulation (Figure 5C,D) and intracellular TC and TG levels (Figure 5E,F) after 12 h. Interestingly, inhibition of IL-6 and TNF-α expression occurred as early as 6 h after OGM treatment (Figure 5G,H), suggesting that GPR68 may influence lipid metabolism through inflammatory pathways.

### 3.6. GPR68 Inhibition Mitigates MASH Development in Mice

**Figure 6:**
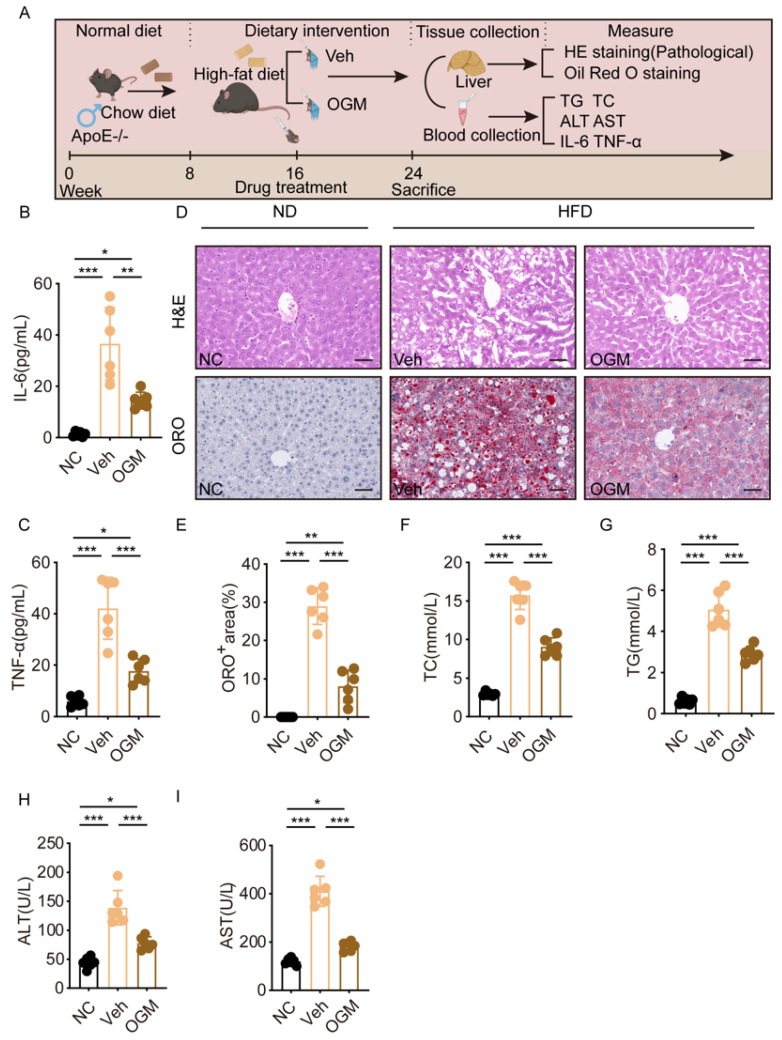
The GPR68 inhibitor OGM attenuated HFD-induced MASH pathology. (**A**) Schematic diagram of OGM (30 mg/kg) treatment in HFD-induced MASH mice. (**B**–**I**) OGM treatment in HFD-induced MASH mice. (**B**) IL-6 levels in mouse serum were determined. (**C**) TNF-α levels in mouse serum were determined. (**D**) Representative H&E and Oil Red O staining of mouse liver tissue, scale bar, 50 μm. (**E**) Quantification of ORO-positive areas in liver tissue from mice. (**F**–**I**) Serum biochemical test. The levels of (**F**) TC, (**G**) TG, (**H**) ALT, (**I**) AST. Data are presented as mean ± SEM. (**B**–**I**) *n* = 6 per group. One-way ANOVA followed by Tukey’s test for (**B**,**C**,**E**–**I**), * *p* < 0.05, ** *p* < 0.01, *** *p* < 0.001.

In vitro experiments suggest that inhibiting GPR68 alleviates hepatocyte inflammation and lipid accumulation. Therefore, we further investigated its role in regulating MASH progression in vivo. Based on preliminary dose-ranging experiments, we established an HFD-induced MASH model and delivered OGM (30 mg/kg) via oral gavage during induction to assess its impact on disease progression (Figure 6A). First, serum IL-6 and TNF-α levels were measured by ELISA. The results showed that OGM treatment significantly reduced the serum IL-6 and TNF-α levels, suggesting that GPR68 inhibition mitigates systemic inflammation associated with MASH (Figure 6B,C). Consistently, following OGM intervention in HFD-fed mice, HE and Oil Red O staining demonstrated improved hepatic histology along with reduced steatosis (Figure 6D,E). Serum TC and TG levels were concurrently reduced (Figure 6F,G). These findings suggest that GPR68 inhibition effectively reduces hepatic lipid deposition and systemic lipid burden in MASH mice. Liver injury is one of the core phenotypes of MASH. We found that OGM treatment also reduced the serum ALT and AST levels, indicating that GPR68 inhibition alleviates MASH-associated liver injury (Figure 6H,I).

Collectively, these data identify GPR68 as a key driver of MASH and a promising therapeutic target.

## 4. Discussion

MASH is a progressive metabolic and inflammatory disease characterized by hepatic steatosis with systemic manifestations and substantial long-term morbidity, yet current therapeutic options remain limited and incompletely address inflammatory drivers of disease progression [17,18,19,20,21]. In this study, we demonstrate that the proton-sensing GPCR GPR68 plays a previously underappreciated role in coordinating hepatic inflammatory and metabolic responses in experimental models of MASH. While the inflammatory effects of GPR68 inhibition are consistent with prior reports implicating this receptor in pH-dependent inflammatory signaling, our findings further suggest that GPR68 may also influence hepatocyte-intrinsic lipid metabolism. The concurrent reduction in inflammatory mediators and intracellular lipid accumulation indicates a close coupling between GPR68-driven inflammatory signaling and metabolic dysregulation in MASH, providing a mechanistic framework in which GPR68 functions as an integrative node linking microenvironmental stress, inflammation, and lipid homeostasis.

**Figure 7:**
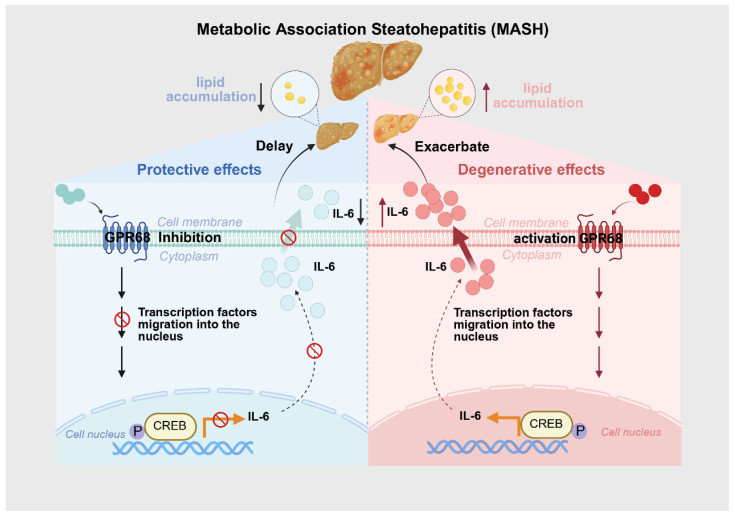
Mechanistic illustration of GPR68 receptor function in the context of Metabolic Associated Steatohepatitis (MASH). The role of GPR68 receptor activation and inhibition in the progression of MASH. The left panel depicts the protective effects of GPR68 inhibition, where the receptor blocks transcription factors from entering the nucleus, leading to reduced IL-6 production and attenuated MASH progression. The right panel shows degenerative effects of GPR68 activation, promoting transcription factor migration into the nucleus and increasing IL-6 levels, potentially exacerbating MASH. The liver illustrations indicate the progression of the disease with GPR68 activation.

Notably, the upregulation of GPR68 occurred under conditions mimicking metabolic overload and inflammatory stress, supporting its pathophysiological relevance rather than a nonspecific stress response. Increased GPR68 expression was observed together with elevated IL-6 levels, suggesting a potential role of GPR68 in inflammatory signaling during MASH progression. Importantly, our data indicated that inflammatory activation preceded overt lipid accumulation in hepatocytes, supporting the notion that inflammation may act as an upstream driver of metabolic dysregulation rather than a secondary consequence of steatosis. The concurrent attenuation of inflammatory signaling and lipid accumulation following GPR68 inhibition supports a model in which GPR68 functions as an integrative signaling node linking microenvironmental stress to both inflammatory and metabolic pathways. Based on previous studies, GPCR-associated signaling cascades can converge on the transcription factor CREB via pathways such as cAMP/PKA. Acting as a signaling integrator, CREB regulates the transcription of inflammation-related genes, including IL-6, across diverse physiological and pathological contexts. Accordingly, the inclusion of CREB in the schematic model is supported by existing literature and is intended to represent a downstream transcriptional integrator rather than a direct mechanistic conclusion of the present study [22,23,24]. This dual regulatory role distinguishes GPR68 from passive inflammatory markers and positions it as an active contributor to MASH pathogenesis. A schematic summary of this proposed mechanism is provided in Figure 7.

To understand how GPR68 contributes to inflammatory amplification during MASH progression, we next focused on its established role as a sensor of extracellular acidification. Mechanistically, GPR68 functions as a sensor of acidic microenvironments and is activated under extracellular acidification conditions [25,26], a hallmark increasingly recognized in metabolically inflamed tissues. Hepatic acidification may arise from hypoxia, mitochondrial dysfunction, lactate accumulation, and altered fatty acid oxidation during MASH progression, creating a permissive niche for pH-sensitive signaling. Such microenvironmental changes are increasingly viewed as active regulators of disease rather than secondary byproducts of metabolic stress. Local tissue acidification has been reported in fatty liver disease models, including AGK-deficient mice exhibiting typical MASH features [8], further supporting this concept. Consistent with previous observations that extracellular acidification induces rapid IL-6 transcription via GPR68 signaling [27], we show that GPR68 activation enhances inflammatory responses associated with IL-6 under steatotic conditions. Notably, IL-6 induction preceded detectable lipid accumulation, suggesting that inflammatory activation may represent an early event driving metabolic dysregulation rather than a mere consequence of steatosis. This temporal sequence aligns with prior studies demonstrating that IL-6 suppresses lipolysis, alters lipid handling, and promotes lipid storage, thereby exacerbating hepatic steatosis [28,29,30,31]. Together, these data support a feed-forward model in which acidic stress activates GPR68, amplifies inflammatory signaling, and secondarily disrupts hepatic lipid metabolism.

Beyond its disease-specific role revealed in this study, the identification of GPR68 as an active driver of MASH has important implications for therapeutic development. Our findings position GPR68 within the broader context of GPCRs, which represent one of the most druggable protein families and account for approximately one-third of FDA-approved therapeutics [32,33]. Increasing evidence indicates that GPCRs play critical roles in metabolic tissues, including the liver, gut, adipose tissue, and pancreatic islets, and serve as effective therapeutic targets for obesity, diabetes, and fatty liver disease [34,35,36]. In this regard, our data extend this paradigm by identifying a proton-sensing GPCR as a regulator of hepatic inflammation and metabolic dysregulation in MASH. While this class of receptors, including GPR68, has previously been implicated in tumor progression, intestinal inflammation, bone metabolism, and respiratory disorders [37,38,39,40], their contribution to hepatic inflammatory disease has remained largely unexplored. Our findings therefore expand the functional repertoire of these receptors and identify GPR68 as a previously unrecognized mediator linking microenvironmental acidification to inflammatory and metabolic signaling pathways that drive MASH progression.

From a therapeutic perspective, the small-molecule GPR68 inhibitor OGM demonstrated robust efficacy in reducing inflammation, steatosis, and liver injury markers in preclinical models. OGM is thought to act as an allosteric inhibitor of GPR68, a receptor capable of engaging both the Gαq–PLC–Ca^2+^ and Gαs–cAMP–PKA signaling pathways. Inhibition of these pathways has been shown to attenuate pro-inflammatory signaling cascades, including NF-κB and AP-1 activation, which are key drivers of IL-6 transcription [41,42,43,44]. In addition to inflammatory responses mediated by cytokines, emerging evidence suggests that proton-sensing GPCRs, including GPR68, may also intersect with inflammatory pathways driven by lipid signaling. In particular, activation of GPR68 under conditions of extracellular acidification has been reported to promote COX-2 expression and prostaglandin production through Gq/PLC/Ca^2+^-dependent signaling, providing a mechanistic link between GPR68 signaling and the eicosanoid pathway derived from arachidonic acid [7]. Although these lipid mediators were not directly assessed in the present study, this pathway may represent an additional inflammatory dimension regulated by GPR68 and warrants further investigation. The ability to concurrently modulate multiple downstream signaling axes may be particularly advantageous in complex diseases such as MASH, where redundant inflammatory pathways contribute to disease persistence.

In the present study, IL-6 and TNF-α were selected as representative pro-inflammatory cytokines, as they are well-established markers of hepatic inflammation and disease activity in MASH. While additional inflammatory mediators may also contribute to the inflammatory milieu, the observed modulation of these core cytokines suggests that GPR68 contributes to inflammatory regulation in the context of MASH. HepG2 cells were used as an in vitro hepatocellular model because they retain key metabolic and inflammatory signaling pathways relevant to lipid accumulation and cytokine responses, and they are widely used in studies investigating hepatic steatosis and drug responses. This model allows for the controlled assessment of hepatocyte-intrinsic effects of GPR68 modulation independent of systemic influences. At present, a direct quantitative comparison of GPR68 expression between HepG2 cells and primary human hepatocytes has not been systematically established. It should also be noted that GPR68 has been reported to be broadly expressed in various human tumors and tumor-derived cell lines [37,44]. As HepG2 cells are derived from hepatocellular carcinoma, tumor-associated alterations in GPR68 expression or signaling context may influence the magnitude or characteristics of GPR68-dependent responses observed in this model. Therefore, the in vitro findings obtained from HepG2 cells should be interpreted as reflecting hepatocyte-associated signaling within a transformed cellular context, rather than fully representing normal hepatocyte physiology. Importantly, the overall consistency between the in vitro HepG2 findings and the in vivo results obtained in diet-induced MASH mouse models supports the relevance of GPR68 signaling in hepatic inflammation beyond strictly tumor-specific contexts.

In OA/PA-induced HepG2 steatosis models, OGM significantly reduced inflammatory mediator secretion and lipid accumulation, indicating a direct hepatocellular effect independent of systemic influences. The observed reduction in lipid accumulation following GPR68 inhibition may be explained by the close interplay between inflammatory signaling and hepatic lipid metabolism. Pro-inflammatory cytokines such as IL-6 and TNF-α are known to promote de novo lipogenesis, impair fatty acid β-oxidation, and exacerbate hepatocellular lipid retention. Therefore, suppression of these cytokines may indirectly alleviate lipid accumulation. In addition, GPR68 is capable of coupling to the Gαq–Ca^2+^ and Gαs–cAMP signaling pathways, which have been reported to interface with key metabolic regulators involved in lipid homeostasis, including AMPK, SREBP-1c, and PPARα. Although these metabolic pathways were not directly examined in the present study, the reduction in lipid accumulation observed in OA/PA-treated HepG2 cells suggests a potential hepatocyte-intrinsic metabolic role of GPR68 inhibition beyond its anti-inflammatory effects. In vivo, OGM treatment alleviated HFD-induced hepatic steatosis, inflammatory infiltration, and elevated serum ALT/AST levels, supporting its efficacy in a physiologically relevant disease context. The dosing regimen (30 mg/kg, once daily) was selected based on preliminary feasibility and efficacy assessments (Supplementary Figure S2), which demonstrated robust anti-inflammatory and hepatoprotective effects without overt toxicity, thereby validating GPR68 inhibition as a viable therapeutic strategy.

Importantly, genetic studies indicate that the global deficiency of GPR68 in mice is compatible with normal development and survival and does not cause overt physiological abnormalities under basal conditions, despite context-dependent roles in specific tissues [45,46]. In parallel, emerging pharmacological and mechanistic studies indicate that GPR68 represents a functionally relevant and pharmacologically accessible target in disease models, supporting its potential therapeutic tractability [47]. These observations suggest that transient or tissue-targeted GPR68 inhibition may be therapeutically feasible with an acceptable safety margin, particularly in chronic inflammatory settings where long-term tolerability is critical. Nevertheless, existing studies have largely focused on receptor pharmacology and disease-relevant efficacy, while comprehensive pharmacokinetic profiling, including bioavailability, tissue distribution, and elimination half-life, has not yet been systematically reported [40,48]. In addition, dose–response relationships and potential effects of chronic administration were not fully explored in this study. Addressing these issues through dedicated pharmacokinetic, pharmacodynamic, and long-term toxicity studies will be essential for translational development and for defining optimal therapeutic windows in chronic metabolic liver disease.

Several limitations should be acknowledged. First, systemic GPR68 inhibition precludes a definitive attribution of observed effects to specific hepatic cell populations, such as hepatocytes, Kupffer cells, or hepatic stellate cells, particularly given the reported cell type-dependent biased signaling of GPR68 [47,49,50,51]. Dissecting these cell-specific contributions using conditional-knockout models or cell-targeted delivery approaches will be essential to fully elucidate the mechanisms underlying GPR68-mediated disease modulation. Second, the in vitro experiments relied on HepG2 cells, a hepatocellular carcinoma-derived cell line, which may not fully recapitulate the phenotype and signaling context of primary human hepatocytes. Third, the potential contribution of related proton-sensing receptors, including GPR4 and GPR65, was not directly examined. Although their expression changes were minimal in our models, compensatory or synergistic effects under acidic conditions cannot be excluded. Fourth, as with most preclinical studies, murine models do not fully recapitulate the genetic heterogeneity, fibrosis kinetics, and metabolic comorbidities of human MASH [52], highlighting the need for validation in human-derived systems such as liver organoids, precision-cut liver slices, or patient-based cohorts.

## 5. Conclusions

In conclusion, this study identifies GPR68 as a key proton-sensing regulator linking tissue acidification to IL-6-driven inflammation and metabolic dysfunction in MASH. By integrating microenvironmental sensing with inflammatory and metabolic signaling, GPR68 occupies a central position in disease progression and represents a mechanistic bridge between metabolic stress and immune activation. Targeting GPR68 effectively attenuates hepatic inflammation and lipid accumulation in preclinical models, highlighting its therapeutic potential beyond conventional lipid-centric approaches. While further studies are required to refine cell-specific mechanisms, optimize pharmacological properties, and establish long-term safety, our findings expand the therapeutic landscape of metabolic dysfunction-associated steatotic liver disease and support GPR68 as a promising target for future MASH interventions aimed at simultaneously addressing inflammatory and metabolic disease components.

## Data Availability

All data supporting the conclusions of this study are included in this paper.

## Supplementary Materials

The following supporting information can be downloaded at: https://www.mdpi.com/article/10.3390/biology15030233/s1, Supplementary Figure S1. Original Western blot images used for quantification. Figure S2. Dose screening of the GPR68 inhibitor OGM and assessment of its toxicity were conducted. (A) Mouse weight changes over three days. (B) Mouse survival rate within three days. (C) ALT content in the culture medium. (D) AST content in the culture medium. (E) IL-6 levels in cell culture supernatant. (F) TNF-α levels in cell culture supernatant. Data are presented as mean ± SEM. (A,B) *n* = 3. (C–F) *n* = 4. Two-way ANOVA followed by Dunnett’s test for (A), two-way ANOVA followed by Tukey’s test for (B–F). * *p* < 0.05, ** *p* < 0.01, *** *p* < 0.001. ns, not significant. Supplementary Table S1. Primer sequences used for RT-qPCR analysis. Supplementary Table S2. Densitometric analysis of Western blot bands quantified by ImageJ.

## Abbreviations

ALT	Alanine aminotransferase
AST	Aspartate aminotransferase
GPCRs	G protein-coupled receptors
GPR4	G protein-coupled receptor 4
GPR65	G protein-coupled receptor 65
GPR68	G protein-coupled receptor 68
H&E	Hematoxylin and eosin
HFD	High-fat diet
MASH	Metabolic dysfunction-associated steatohepatitis
OA	Oleic acid
OGM	Ogremorphin
PA	Palmitic acid
RT-qPCR	Quantitative reverse transcription polymerase chain reaction
TC	Total cholesterol
TG	Triglycerides
WB	Western blot
