# Supplementary material for: Targeting GPR68 Alleviates Inflammation and Lipid Accumulation in Metabolic Dysfunction-Associated Steatohepatitis
Source: Biology (Basel). 2026 Jan 26;15(3):233. doi: 10.3390/biology15030233 (PMC12896580; doi:10.3390/biology15030233)
Supplement: Supplementary file 1 [file biology-15-00233-s001.zip › Supplementary Figure S1.pdf]

Supplementary Figure S1. Original Western blot images used for quantification.

Figure 1N

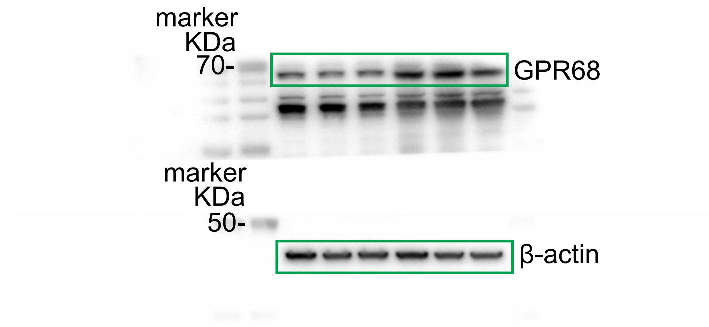

Western blot image corresponding to Figure 1(N),
including molecular weight markers indicated according to the manufacturer’s specifications.

Figure 2K

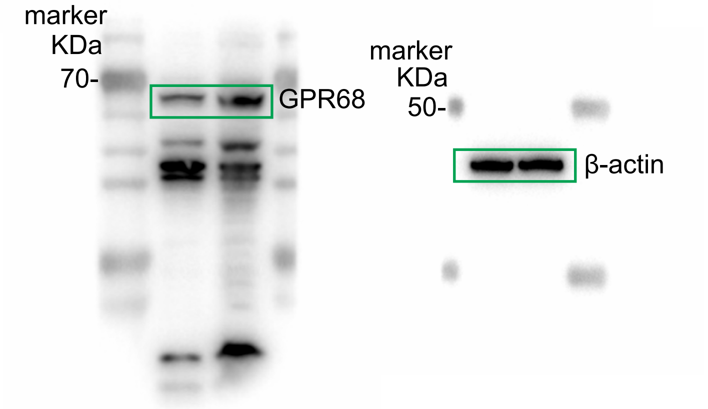

Western blot image corresponding to Figure 2(K),
including molecular weight markers indicated according to the manufacturer’s specifications.

Figure 4A

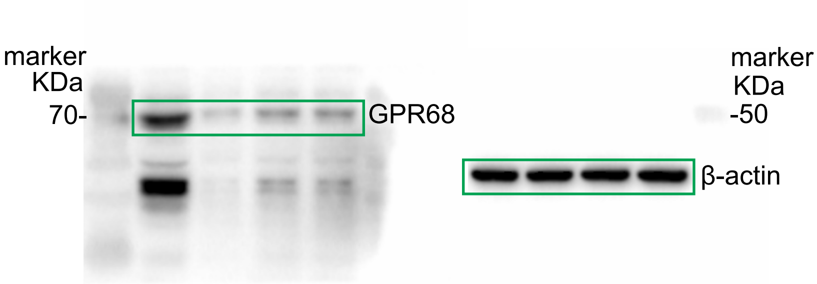

Western blot image corresponding to Figure 4(A),
including molecular weight markers indicated according to the manufacturer’s specifications.
