# Supplementary material for: Targeting GPR68 Alleviates Inflammation and Lipid Accumulation in Metabolic Dysfunction-Associated Steatohepatitis
Source: Biology (Basel). 2026 Jan 26;15(3):233. doi: 10.3390/biology15030233 (PMC12896580; doi:10.3390/biology15030233)
Supplement: Supplementary file 1 [file biology-15-00233-s001.zip › Supplementary Figure S2.pdf]

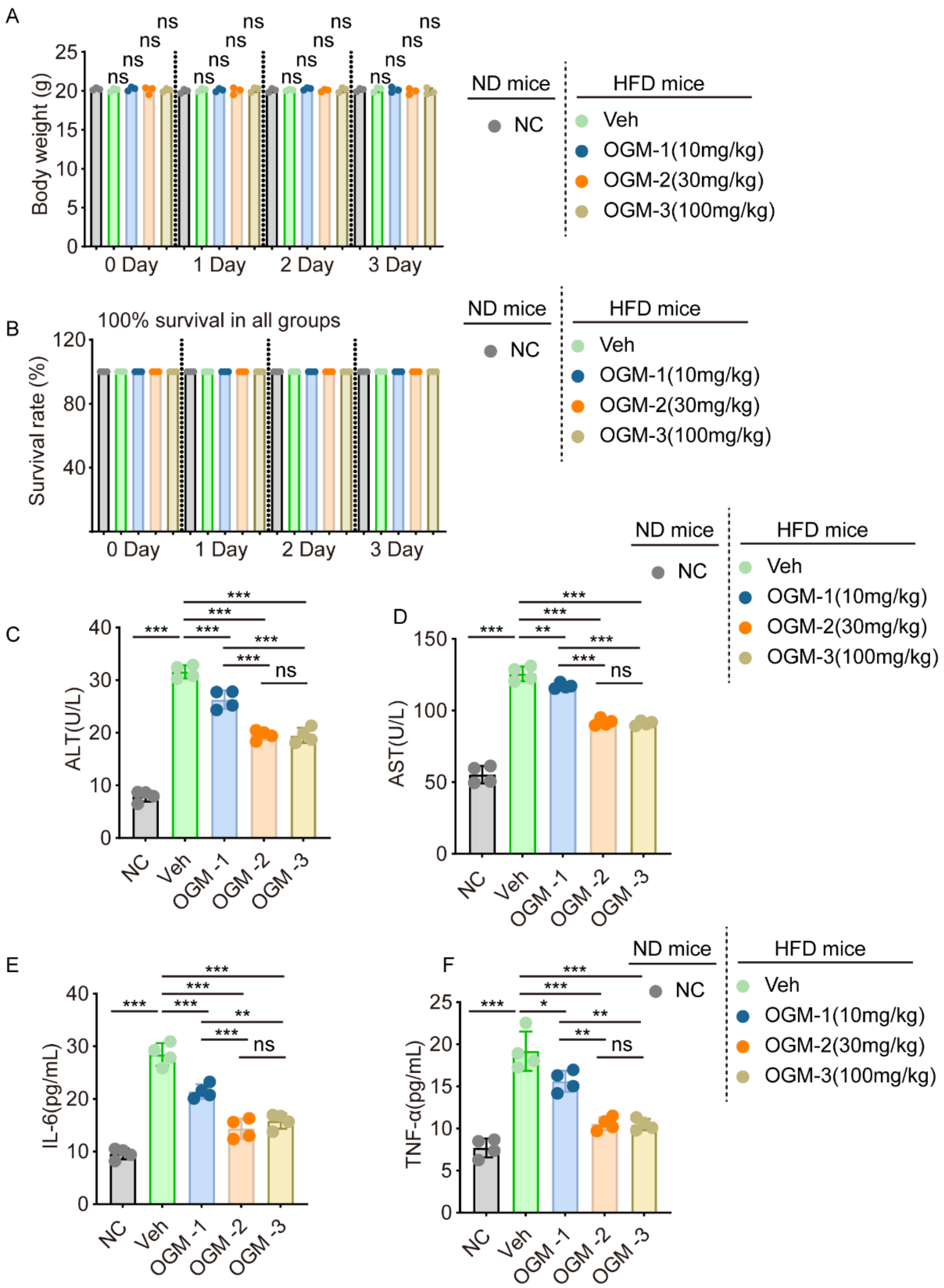

Figure S2: Dose screening of the GPR68 inhibitor OGM and assessment of its toxicity were conducted. (A) Mouse weight changes over three days. (B) Mouse survival rate within three days. (C) ALT content in the culture medium. (D) AST content in the culture medium. (E) IL-6 levels in cell culture supernatant. (F) TNF-α levels in cell culture supernatant. Data are presented as mean ± SEM. (A, B) n=3. (C-F) n=4. Two-way ANOVA followed by Dunnett's test for (A), two-way ANOVA followed by Tukey's test for (B-F). *p < 0.05, **p < 0.01, ***p < 0.001. ns, not significant.
