# Supplementary material for: Targeting GPR68 Alleviates Inflammation and Lipid Accumulation in Metabolic Dysfunction-Associated Steatohepatitis
Source: Biology (Basel). 2026 Jan 26;15(3):233. doi: 10.3390/biology15030233 (PMC12896580; doi:10.3390/biology15030233)
Supplement: Supplementary file 1 [file biology-15-00233-s001.zip › Supplementary Table S1.pdf]

Supplementary Table S1. Primer sequences used for RT–qPCR analysis

A. Mouse primers

Gene	Forward primer (5′–3′)	Reverse primer (5′–3′)	Amplicon (bp)
Gapdh	TCACTGCCACCCAGAAGAC	TGTAGGCCATGAGGTCCAC	450
Gpr68	TATCTTGCCCCATCGACCACA	AGTACCCGAAGTAGAGGGACA	111
Gpr4	GCTGGGCGTCTACCTGATG	AGGCGATGCTGATATAGATGTTG	167
Gpr65	ATGGCGATGAACAGCATGTG	ACGCATAAAGATCCGATGTTGG	119

B. Human primers

Gene	Forward primer (5′–3′)	Reverse primer (5′–3′)	Amplicon (bp)
GAPDH	ACAACAGCCTCAAGATCATCAGCAAT	GTCCTTCCACGATACCAAAGTTGTCA	99
GPR68	TGTACCATCGACCATACCATCC	GGTAGCCGAAGTAGAGGGACA	106
GPR4	CTCTTCCGAGACCGCTACAAC	ACACCCGATAGAGGTTCATCC	88
GPR65	TGATCTGCAACCGGAAAGTCT	TCAGCAACATCACATGAAAGGG	144

Notes

Primer sequences are shown in the 5′–3′ direction.
Annealing temperatures ranged from 58 to 63 °C depending on primer sets.
Mouse gene symbols are written with an initial capital letter followed by lowercase letters, whereas human gene symbols are written in all uppercase.
Primer sequences were obtained from NCBI RefSeq or PrimerBank.
