# Supplementary material for: Targeting GPR68 Alleviates Inflammation and Lipid Accumulation in Metabolic Dysfunction-Associated Steatohepatitis
Source: Biology (Basel). 2026 Jan 26;15(3):233. doi: 10.3390/biology15030233 (PMC12896580; doi:10.3390/biology15030233)
Supplement: Supplementary file 1 [file biology-15-00233-s001.zip › Supplementary Table S2.pdf]

Supplementary Table S2. Densitometric analysis of Western blot bands quantified by ImageJ.

Figure 1 N:

	ND	ND	ND	HFD	HFD	HFD
	1	2	3	1	2	3
GPR68	105256	90121	96495	154504	170280	135234
actin	155073	124232	126932	147593	123124	113559
GPR68/actin	0.678751	0.725425	0.760210	1.046824	1.382996	1.190869

Figure 2 N:

	Veh	OA/PA
GPR68	32449	67562
actin	90795	83785
GPR68/actin	0.357387	0.806373

Figure 4 A:

	siCtrl	SiGPR68-1	SiGPR68-2	SiGPR68-3
GPR68	79832	8174	33622	33122
actin	110134	109008	110899	117868
GPR68/actin	0.72486244	0.074985322	0.303176764	0.281009265
